# Effects of Upper Trapezius Myofascial Trigger Points on Scapular Kinematics and Muscle Activation in Overhead Athletes

**DOI:** 10.2478/hukin-2022-000079

**Published:** 2022-11-08

**Authors:** Lin-Ling Huang, Tsun-Shun Huang, Yang-Hua Lin, Cheng-Ya Huang, Jing-Lan Yang, Jiu-Jenq Lin

**Affiliations:** 1 School and Graduate Institute of Physical Therapy, College of Medicine, National Taiwan University, Taipei, Taiwan.; 2 Department of Physical Therapy and Assistive Technology, National Yang Ming Chiao Tung University, Taipei, Taiwan.; 3 School of Physical Therapy, Chang Gung University, Taoyuan, Taiwan.; 4 Division of Physical Therapy, Department of Physical Medicine and Rehabilitation, National Taiwan University Hospital, Taipei, Taiwan.; 5 Department of Orthopedics, Fu Jen Catholic University Hospital, Fu Jen Catholic University, New Taipei City, Taiwan.

**Keywords:** muscle fatigue, trapezius, scapular posterior tipping

## Abstract

Prolonged overactivity of the upper trapezius muscle with myofascial trigger points might cause muscle fatigue and subsequently change scapular kinematics and associated muscular activities. Scapular kinematics and associated muscular activities were investigated in 17 overhead athletes with upper trapezius myofascial trigger points and 17 controls before and after a fatigue task. Participants performed a fatigue task requiring sustained isometric scapular elevation. The outcomes included scapular kinematics (upward/downward rotation, external/internal rotation, posterior/anterior tilt) that were tracked by the Polhemus FASTRAK (Polhemus Inc., Colchester, VT, USA) system with Motion Monitor software and muscular activities (upper trapezius, lower trapezius and serratus anterior) that were collected at 1000 Hz per channel using a 16-bit analog-to-digital converter (Model MP 150, Biopac systems Inc., CA, USA) with pairs of silver chloride circular surface electrodes (The Ludlow Company LP, Chocopee, MA) during arm elevation. Mixed ANOVAs were conducted to characterize the outcomes with and without a fatigue task in participants with myofascial trigger points. Decreased scapular posterior tipping during 90 degrees of arm raising/lowering (effect sizes of 0.51 and 0.59) was likely to be elicited by the scapular elevation fatigue task in the presence of myofascial trigger points. Activity of the lower trapezius was higher in the myofascial trigger point group (6.2%, p = 0.036) than in the control group. Following the fatigue task, both groups showed increased activity in the upper trapezius (9.0%, p = 0.009) during arm lowering and in the lower trapezius (2.7%, p < 0.01) during arm raising and lowering. Decreased scapular posterior tipping during 90 degrees of arm raising/lowering after a fatigue task may lead to impingement. We found that the presence of upper trapezius myofascial trigger points in amateur overhead athletes was related to impaired scapular kinematics and associated muscular activities during arm elevation after a fatigue task, especially the decreased scapular tipping during 90 degrees of raising/lowering.

## Introduction

Scapular movements are critical for normal function of the upper extremities. Owing to the lack of bony articulations between the trunk and the scapula, the stability and mobility of the scapula depend on the actions of scapular muscles (Cools et al., 2007; Umehara et al., 2018). Since the upper trapezius (UT), lower trapezius (LT) and serratus anterior (SA) muscles work as a force couple for upward rotation of the scapula, any imbalance in their muscular activities may result in deficits of scapular control (Umehara et al., 2018). Inadequate scapular upward rotation (UR), external rotation (ER), and posterior tilting (PT) during arm elevation are associated with scapular muscle imbalance in subjects with shoulder dysfunction (Chopp-Hurley and Dickerson, 2015; Cobanoglu et al., 2021; Ludewig and Cook, 2000). Overhead athletes participating in sports such as baseball, badminton, tennis, volleyball, and swimming frequently perform rapid, repetitive overhead movements with a wide range of motion (Batalha et al., 2013; Drigny et al., 2020). Such movements may place excessive stress on muscles in the shoulder region and even result in their overloading, putting overhead athletes at a high risk of shoulder injuries (Drigny et al., 2020; Umehara et al., 2018).

The myofascial trigger point (MTrP) has been defined as a hypersensitive palpable area in a taut band of skeletal muscle (Donnelly et al., 2018). MTrPs are extremely common and may become a painful component of nearly everyone's lives. The most common location of MTrPs is the UT, with 78.8% of healthy people having latent trigger points (LTrPs) (Lucas et al., 2008). It has been reported that the prevalence of shoulder muscles hosting active TrPs is 78% (infraspinatus), 58% (upper trapezius) and 50% (middle deltoid), while that of LTrPs is 49% (teres major) and 38% (upper trapezius) (Bron et al., 2011). The common clinical characteristics are exquisite tenderness on the taut band, local and/or referred pain, local twitch response, limited range of motion, motor dysfunction, and autonomic phenomena (Donnelly et al., 2018; Fricton, 2016; Gerwin, 2014), as well as electromyographic features of increased responsiveness, delayed relaxation, and increased fatigability (Ge et al., 2012; Hagberg and Kvarnstrom, 1984; Simons et al., 1999).

Previous studies have shown that overhead sports activities tend to place excessive stress on muscles in the shoulder region and thus give rise to muscle overloading and fatigue (Bron et al., 2011; Hidalgo-Lozano et al., 2011). This situation may lead to the development of MTrPs, especially in the UT muscle, which functions as a scapular upward rotator (Lucas et al., 2008). In a population of elite swimmers who execute repetitive overhead shoulder movements, the prevalence of UT muscles harboring MTrPs is estimated to be about 66.7% in healthy swimmers and 88.2% in those with shoulder problems (Hidalgo-Lozano et al., 2011). In addition, MTrPs have been found to alter the activation pattern of shoulder region muscles (Lucas et al., 2004, 2010), especially resulting in UT muscle activity latency during arm elevation movements in people with UT MTrPs (Bohlooli et al., 2016). Given the fact that overloading leads to the appearance of MTrPs, it is not clear how MTrPs influence fatigue tasks.

The purposes of this study were to (1) compare the differences in the scapular kinematics and muscular activities during an arm elevation task between overhead athletes with and without UT MTrPs, and (2) investigate the effects of a scapular elevation fatigue task on the scapular kinematics and muscular activities during arm elevation in overhead athletes with UT MTrPs. The first hypothesis was that subjects with UT MTrPs would have significant increases in UT activity and decreases in the activities of the LT and SA, along with significant decreases in scapular UR, ER and PT when comparing to those without UT MTrPs, which are impingement-sparing changes in scapular kinematics. The second hypothesis was that overhead athletes would have significant decreases in scapular UR, ER and PT due to the dysfunction of the UT muscle, as well as increases in muscular activities (UT, LT and SA) after the scapular elevation fatigue task, especially in subjects with UT MTrPs.

## Methods

### Participants

According to a previous study (Rich et al., 2016), a group size of 17 participants can provide 80% power to detect a 2.5° difference in scapular upward rotation, with an effect size of 0.9. Amateur overhead athletes (baseball, badminton, tennis, volleyball, or swimming) were recruited from sports teams at a university or through local Internet media. All participants provided written informed consent, and the study was approved by the Ethics Committee Institutional Review Board. Subjects were included if they (1) were 18 to 40 years old, and (2) habitually engaged in overhead sports activities for at least 5 hours per week. Athletes were assigned to the MTrP group (MG) if they met the following criteria: (1) taut band of the UT muscle; (2) palpable tender spots in the UT muscle; and (3) pressure pain threshold (PPT) < 2.9 kg/cm^2^ for males and < 2.0 kg/cm^2^ for females, which are the lowest PPTs at which a muscle can be considered “normal” (Fischer, 1987). Subjects meeting less than one of the criteria were assigned to the control group. Subjects were excluded if they (1) had a history of shoulder surgery, and/or (2) took medications for shoulder pain. Thirty-six athletes were screened, and two participants did not meet the inclusion criteria. Thus, the remaining 34 participants were recruited (age: 20.9 ± 2.1 vs. 23.1 ± 2.3; gender: 14 male/3 vs. 12 male/5; BMI: 20.9 ± 1.6 vs. 21.7 ± 1.9) and allocated to one of the two groups (17 participants each).

### Measures

The Polhemus FASTRAK (Polhemus Inc., Colchester, VT, USA) system with Motion Monitor software (Innovative Sports Training Inc., Chicago, IL, USA) was used to track three-dimensional scapular kinematics during motion. The latency was 4 ms with the update rate of 120 updates/s. The accuracy reported by the manufacturer was 0.03” RMS for the X, Y, and Z position and 0.15 degrees RMS for receiver’s orientation. The resolution was 0.0002 inches per inch of transmitter and receiver separation, and 0.025 degrees orientation. The first sensor was placed on the sternum notch, the second was attached to the flat bony surface of the acromion process with adhesive tape, and the third was attached to the distal humerus with Velcro straps. For the local coordinate system, landmarks (the seventh cervical vertebra, eighth thoracic vertebra, twelfth thoracic vertebra, sternal notch, xiphoid process, acromion, anterior glenohumeral joint, posterior glenohumeral joint, root of the spine of the scapula, inferior angle of the scapula, lateral epicondyle, and medial epicondyle) were marked by a physical therapist with a stylus. We followed the International Society of Biomechanics (ISB) guidelines for constructing a shoulder joint coordinate system (Wu et al., 2005). The raw data of the kinematics were low-pass filtered at a 6-Hz cutoff frequency and converted into anatomically-defined rotations. Scapular orientation relative to the thorax was described using an Euler angle sequence of rotation about Zs (internal/external rotation or protraction/retraction), about Y's (downward/upward rotation), and about X"s (posterior/anterior tilting).

The sEMG data were collected at 1000 Hz per channel using a 16-bit analog-to-digital converter (Model MP 150, Biopac systems Inc., CA, USA). Pairs of silver chloride circular surface electrodes (The Ludlow Company LP, Chocopee, MA) with inter-electrode distances (center-to-center) of 10 mm and 20 mm were used for sEMG data collection. The impedance between the electrodes and the skin over the muscle was measured with an impedance meter (Model F-EZM5, Astro-Med Inc., Ri, USA). Impedance of less than 10 kΩ for each electrode placement was required. Recorded signals were amplified with the common-mode rejection ratio (CMRR) of 86 dB at 60 Hz with a bandwidth (-3 dB) of 10 to 500 Hz and Grass AC/DC amplifier (Model 15A12, Astro-Med Inc. RI, USA) gain of 1000 dB. Full bandwidth sEMG data captured by data acquisition software (AcqKnowledge, Biobac systems Inc., CA, USA) were reduced using a root mean square (RMS) algorithm with an effective sampling rate of 50 samples. The mean power frequency (MPF) was the frequency at which the average power within the epoch was reached.

The surface electrodes were placed over the upper trapezius (UT, midway between the acromion and the spinous process of the seventh cervical vertebra), lower trapezius (LT, at the intersection of the spine of the scapula and the spinous process of the seventh thoracic vertebra), and the serratus anterior (SA, anterior to the latissimus dorsi and posterior to the pectoralis major), and a reference electrode was placed on the ipsilateral clavicle. Then maximum voluntary isometric contraction (MVIC) was conducted in the break test. The sEMG signals during the MVIC test were collected for normalization. We collected MVIC before the experiment task to prevent the fatigue effect on the muscle force. The MVIC of the UT was measured during resisted scapular elevation with a load cell, with the resistance applied to the participant’s shoulder by a belt (Kendall et al., 1993). For measurement of the MVIC of the LT, the participant was placed in a prone position with the arm abducted in line with the muscle fibers. Resistance was applied to prevent further elevation. For measurement of the MVIC of the SA muscle, the participant was seated with the arm elevated at 135 degrees. Resistance was applied to the distal upper arm (Kendall et al., 1993). The MVICs were collected for 5 s in each of 2 trials, with a 1-min rest interval in between. The average EMG data of the 5 s MVIC were used for normalization. A hand-held event timer switch was used to mark the beginning of the arm elevation movement, the highest degree of elevation, and the end of the movement.

A digital pressure algometer (Force Ten™ FDX Digital Force Gauge, Wagner Instruments, Greenwich CT, USA) was used to measure the pressure pain threshold (PPT) of the MTrPs. One load cell (BHL ONLINE, Kaohsiung, Taiwan) with two belts was used to measure the peak force of scapular elevation (shoulder shrug), as well as the real-time force feedback of the scapular elevation fatigue protocol. Three trials of bilateral arm elevation in the scapular plane were used for pre-fatigue and post-fatigue outcome measurements. The mean EMG data were recorded during 0–30°, 30–60°, 60–90°, 90–120° and > 120° of arm raising and lowering. The EMG data for each muscle from the three trials were averaged. The mean sEMG amplitude of each muscle was reported as a percentage of MVIC. Full bandwidth sEMG data captured by data acquisition software (AcqKnowledge, Biopac systems Inc., CA, USA) were reduced using a root mean square (RMS) algorithm to produce sEMG envelopes with a sampling rate of 50 samples.

### Design and procedures

This was a cross-sectional study. The independent variables included the two groups (amateur overhead athletes with and without UT MTrPs) and the two conditions (before and after the fatigue task). The dependent variables were the EMG activity of the three muscles and the scapular kinematics. Throughout the experiment, male participants were asked to remove their shirts and female participants to wear a halter top. The pressure pain threshold (PPT) was tested twice by a blinded assessor with a digital algometer, with a 1-min rest interval between each trial. The tip of the algometer was placed perpendicularly on the skin of the dominant-side UT muscle MTrP. The participants were asked to say “ouch” or “pain” immediately when the sensation of pressure turned into pain. At that moment, the value of force was recorded in units of kg-force (kgf) and represented the PPT.

**Figure 1:**
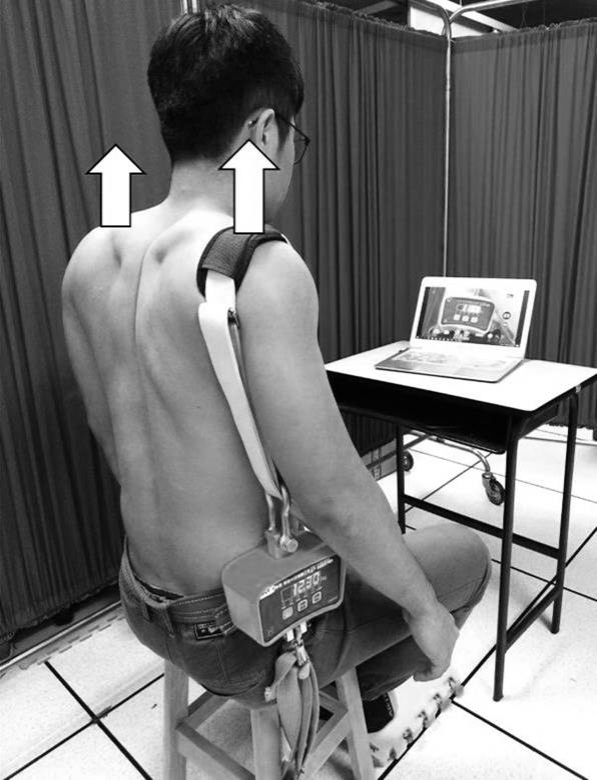
Scapular elevation fatigue task with real-time force feedback. A load cell attached with two orthopedics belts was placed on the dominant side shoulder of the participant, while the other end of the belt was fixed to the chair.

After the placement of the sensors and electrodes as described in the measures section, the arm elevation task was performed. Participants raised their arms in the scapular plane to 180 degrees within 3 s and returned to the starting position within 3 s for three trials, with 1-min rest intervals. Participants were allowed to practice three times to ensure familiarity with the task. Following the arm elevation task, the isometric scapular elevation fatigue task was performed (Figure 1). During the task, compensatory movements such as trunk flexion, rotation, side bending, leaning backward, and hand support on the thigh were not allowed. The main assessor continuously monitored the posture of the participants from the coronal plane and provided verbal encouragement. When the force that participants could hold fell below 50% of the peak force for at least 5 s, the fatigue protocol was terminated. The score of the Borg CR-10 scale at the end of the task and the mean power frequency (MPF) of the UT muscle in the initial and final 5 s of the protocol were collected to quantify the subjective and objective fatigue levels. The Borg Rating of Perceived Exertion (RPE) scale, developed by Swedish researcher Gunnar Borg (Borg, 1982), is a tool for measuring an individual’s effort and exertion, breathlessness and fatigue during physical work. The individual is asked to circle or tick the number that best describes his RPE from zero “no exertion at all” to 10 “maximal exertion”. Immediately upon completing the fatigue protocol, participants performed the task of three scapular-plane arm elevations.

### Statistical analysis

The Statistical Package for the Social Sciences (SPSS) 20.0 was used for data analysis. The Shapiro-Wilk test was performed to confirm normal distribution of all outcome measurements. Independent *t* tests were conducted to check for demographic differences between groups, and non-parametric analysis was used if the data were not normally distributed. For the first purpose of the present study, two-way mixed ANOVA was conducted to analyze the scapular kinematics and muscular activity with factors of group and the arm elevation angle (30°, 60°, 90°, and 120° in arm raising and lowering; muscular activities at 0–30°, 30–60°, 60–90°, 90–120°, and > 120° in arm raising and lowering). For the outcome data which were not normally distributed, nonparametric tests (Wilcoxon Signed Rank test or Mann–Whitney U test) were used to compare the between-group differences. For the second purpose, three-way mixed ANOVA was conducted for scapular kinematics and muscular activity outcomes with factors of fatigue condition (pre-fatigue/post-fatigue), the group and the arm elevation angle (same as above). Alpha was set at 0.05 or adjusted to 0.05 divided by 5 for multiple comparisons as necessary.

## Results

The baseline data were similar between the groups except for the pressure pain threshold (1.1 ± 0.5 vs. 2.6 ± 1.6, *p* < 0.01). There were no differences in the Borg rating scale or duration of the fatigue protocol between the two groups (8.3 ± 1.2 vs. 8.4 ± 1.5, *p* > 0.05; 253.7 ± 108.9 vs. 204.4 ± 92.5 s, *p* > 0.05). Since the minimal decline in MPF indicating local muscle fatigue development is considered to be 8% (Noguchi et al., 2013; Umehara et al., 2018; Zanca et al., 2016), fatigue was validated on the decline of MPF in the UT muscle (28.0% in MTrP vs. 18.8% in control) and LT muscle (18.8% in MTrP).

Two-way mixed ANOVA revealed no interaction or group effect in scapular kinematics. Based on the analysis of two-way mixed ANOVA, activity of the LT had no interaction, but group effect with significantly higher in the MTrP group than in the control group (5.47%, *p* = 0.036). Additionally, there was no interaction or group difference in muscle activity of the UT and SA.

Three-way mixed ANOVA had interaction and indicated that the fatigue protocol had a small to moderate effect size on scapular posterior tipping in the MTrP group (mean effect size = 0.35, range = 0.14–0.59) (Table 1). In the three-way mixed ANOVA test of the UT muscle, UT muscular activity had interaction and was significantly higher after the scapular elevation fatigue protocol during arm lowering of 120–90° (9.0% MVIC, *p* = 0.009) (Table 2). Results of the Wilcoxon Signed Rank test indicated that UT muscular activity was higher at arm lowering angles of > 120° (9.1% MVIC, *p* = 0.007). For the LT muscle activity, significantly higher activity of the LT after the scapular elevation fatigue task was found for arm raising of 0–30° (1.5% MVIC, *p* < 0.0005), 30–60° (4.4% MVIC, *p* < 0.0005), 60–90° (4.7% MVIC, *p* = 0.002), and > 120° (5.9% MVIC, *p* = 0.001), as well as for arm lowering of > 120° (4.5% MVIC, *p* = 0.002) and 30–0° (0.8% MVIC, *p* = 0.005). The Wilcoxon Signed Rank test indicated higher LT activity during arm raising of 90–120° (5.2% MVIC, *p* = 0.003) and during arm lowering of 120–90° (3.8% MVIC, *p* = 0.001) and 90–60° (3.0% MVIC, *p* = 0.002) after the fatigue protocol.

**Table 1:** Scapular kinematics in two groups (mean ± standard deviation, unit: degrees).

	Raising 0–30°	Raising 30–60°	Raising 60–90°	Raising 90–120°	Fatigue effect size
	MTrP group				
UR Pre-F	14.4 ± 3.3	28 ± 4.3	40.8 ± 6.8	52.5 ± 9.9	
UR Post-F	14.2 ± 3.2	28.8 ± 4.9	42.4 ± 7.4	56.8 ± 11.1	0.22(0.11–0.42)
ER Pre-F	5.5 ± 4.0	8.2 ± 6.1	7.7 ± 9.5	4.9 ± 14.8	
ER Post-F	5.2 ± 3.0	7.5 ± 4.8	7.2 ± 7.7	6.7 ± 14.3	0.01(0.01–0.12)
PT Pre-F	5.4 ± 2.9	8.0 ± 5.1	11.1 ± 7.2	15.7 ± 9.8	
PT Post-F	5.0 ± 2.8	7.1 ± 5.5	7.8 ± 7.3*	11.0 ± 10.8	0.35(0.14–0.59)
	Control group				
UR Pre-F	13.6 ± 3.4	24 ± 12.4	35.3 ± 20.3	53.4 ± 6.9	
UR Post-F	12.5 ± 3.3	27.9 ± 4.2	43.4 ± 4.9	56.6 ± 6.7	0.29(0.18–0.47)
ER Pre-F	4.7 ± 2.3	8.1 ± 4.0	10.9 ± 7.8	10.6 ± 18.7	
ER Post-F	4.6 ± 3.1	7.1 ± 4.8	9.2 ± 8.2	10.5 ± 18.1	0.11(0.01–0.22)
PT Pre-F	4.5 ± 2.3	8.1 ± 4.6	13.5 ± 8.1	18.3 ± 10.9	
PT Post-F	4.0 ± 2.4	6.7 ± 4.3	11.1 ± 7.8	17.2 ± 14.9	0.24(0.10–0.44)
	Lowering 120°	Lowering 90°	Lowering 60°	Lowering 30°	
	MTrP group				
UR Pre-F	49.2 ± 10.4	39.8 ± 8.8	27.9 ± 6.2	12.5 ± 5.5	
UR Post-F	53.6 ± 12.1	39.9 ± 7.6	27.4 ± 4.7	11.1 ± 5.2	0.22(0.11–0.42)
ER Pre-F	4.6 ± 14.4	8.3 ± 9.7	10.1 ± 7.3	7.8 ± 4.8	
ER Post-F	5.8 ± 14.1	8.3 ± 8.3	9.3 ± 6.0	7 ± 4.8	0.01(0.01–0.12)
PT Pre-F	15.7 ± 10.1	11.8 ± 7.0	7.2 ± 5.4	3.3 ± 2.1	
PT Post-F	11.9 ± 10.1	8.5 ± 5.7	5.3 ± 4.5	2.9 ± 3.1	0.35(0.14–0.59)
	Control group				
UR Pre-F	50.2 ± 6.9	39 ± 5.3	26.7 ± 4.2	11 ± 3.6	
UR Post-F	53.2 ± 8.2	38.9 ± 6.0	25.4 ± 5.4	9.8 ± 4.1	0.29(0.18–0.47)
ER Pre-F	6.8 ± 18.8	8.5 ± 9.7	9.5 ± 5.7	7.3 ± 3.9	
ER Post-F	7.4 ± 18.4	8.2 ± 8.9	8.7 ± 6.2	6.7 ± 5.1	0.11(0.01–0.22)
PT Pre-F	18.7 ± 13.1	13.2 ± 6.5	8.0 ± 4.8	4.1 ± 3.8	
PT Post-F	15.1 ± 13.8	11.7 ± 6.2	7.5 ± 4.3	2.5 ± 3.1	0.24(0.10–0.44)

F: fatigue task; MTrP: myofascial trigger point; UR: upward rotation; ER: external rotation; PT: posterior tipping; no interaction or group effect in scapular kinematics

**Table 2:** Muscle activity presented as percentage of maximum voluntary isometric contraction in the two groups (mean ± standard deviation).

	Raising 0–30°	Raising 30–60°	Raising 60–90°	Raising 90–120°	Raising > 120°
	MTrP group				
UT Pre-F	20.5 ± 8.4	42.7 ± 17.6	47.9 ± 26.1	61.5 ± 42.7	86.3 ± 59.6
UT Post-F	19.2 ± 11.1	41.4 ± 22.3	50.2 ± 30.7	67.1 ± 41.0	92.4 ± 54.7
LT Pre-F	5.3 ± 2.9*^†^	12.3 ± 7.7*^†^	18.5 ± 13.0*^†^	25.5 ± 17.0*^†^	27.9 ± 18.5*^†^
LT Post-F	7.2 ± 4.1*^†^	16.6 ± 9.7*^†^	23.0 ± 12.7*^†^	31.2 ± 18.4*^†^	32.6 ± 18.0*^†^
SA Pre-F	6.8 ± 2.6	15.7 ± 6.9	19.8 ± 6.9	33.1 ± 14.1	49.6 ± 22.6
SA Post-F	7.0 ± 3.4	14.8 ± 8.0	20.0 ± 8.7	34.9 ± 17.1	52.3 ± 26.0
	Control group				
UT Pre-F	24.4 ± 16.7	46.5 ± 30.5	49.3 ± 34.0	59.0 ± 48.3	71.3 ± 80.3
UT Post-F	22.0 ± 14.5	46.1 ± 31.6	60.2 ± 51.7	74.0 ± 65.3	83.6 ± 73.6
LT Pre-F	4.9 ± 3.0*^†^	8.1 ± 4.8*^†^	12.2 ± 8.2*^†^	17.3 ± 11.3*^†^	19.7 ± 14.2*^†^
LT Post-F	5.3 ± 2.6*^†^	13.5 ± 7.9*^†^	17.2 ± 9.3*^†^	21.9 ± 12.5*^†^	26.1 ± 16.6*^†^
SA Pre-F	7.4 ± 3.1	15.2 ± 7.2	22.2 ± 5.7	35.4 ± 16.4	47.5 ± 26.9
SA Post-F	7.0 ± 2.8	15.2 ± 7.2	24.7 ± 11.6	38.6 ± 17.7	51.8 ± 26.9
	Lowering > 120°	Lowering 120– 90°	Lowering 90–60°	Lowering 60–30°	Lowering 30–0°
	MTrP group				
UR Pre-F	51.8 ± 27.9**^†^**	35.3 ± 18.4**^†^**	27.7 ± 13.9	18.2 ± 8.9	9.3 ± 6.1
UR Post-F	57.6 ± 32.0**^†^**	43.1 ± 24.2**^†^**	34.7 ± 21.9	19.0 ± 11.9	8.2 ± 7.0
ER Pre-F	22.8 ± 17.3*^†^	15.7 ± 12.1*^†^	12.5 ± 10.0*^†^	8.8 ± 6.5*	4.4 ± 3.0*^†^
ER Post-F	26.4 ± 15.3*^†^	19.1 ± 10.4*^†^	14.6 ± 7.6*^†^	10.0 ± 4.8*	5.2 ± 3.5*^†^
PT Pre-F	31.7 ± 14.1	14.5 ± 4.8	8.8 ± 3.3	5.0 ± 2.2	3.9 ± 1.9
PT Post-F	32.5 ± 12.9	17.1 ± 7.5	9.2 ± 4.6	5.2 ± 2.6	3.4 ± 1.9
	Control group				
UR Pre-F	42.4 ± 32.7	31.3 ± 22.6	27.2 ± 19.0	20.5 ± 15.0	10.3 ± 8.1
UR Post-F	54.8 ± 43.9	41.5 ± 37.7	32.0 ± 28.2	17.9 ± 14.7	7.8 ± 6.2
ER Pre-F	13.1 ± 10.4*^†^	8.4 ± 6.5*^†^	6.7 ± 4.5*^†^	5.3 ± 3.7*	3.3 ± 1.3*^†^
ER Post-F	18.3 ± 10.3*^†^	12.5 ± 6.5*^†^	10.6 ± 6.3*^†^	7.4 ± 4.4*	4.1 ± 1.9*^†^
PT Pre-F	33.0 ± 18.9	19.7 ± 8.3	11.9 ± 5.2	7.2 ± 3.5	3.9 ± 1.6
PT Post-F	35.0 ± 17.5	20.9 ± 8.5	11.1 ± 5.3	6.2 ± 3.1	4.2 ± 2.0

F: fatigue task; MTrP: myofascial trigger point; ^*^ significant difference between two groups; ^††^ significant difference between pre-fatigue and post-fatigue (^†^Wilcoxon Signed Rank test)

## Discussion

The present study investigated the influence of UT MTrPs as well as the effects of scapular elevation fatigue on scapular kinematics and associated muscular activities in overhead athletes during arm elevation in the scapular plane. To our knowledge, this is the first study to investigate the differences in scapular kinematics in overhead athletes with and without UT MTrPs, and to examine the effects of upper trapezius muscle fatigue in these subjects with an elevation fatigue task.

Based on the results of the current study, we believe that the presence of UT MTrPs is related to scapular posterior tipping impairment following a fatigue protocol in overhead athletes. Despite the lack of significant differences in scapular kinematics between participants with and without UT MTrPs, the effect size demonstrated that overhead athletes with UT MTrPs had decreased scapular posterior tipping (effect sizes: 0.59 and 0.52 during arm raising and lowering of 90 degrees, respectively). Type II error in our results was likely, as 30–38% power was calculated based on 17 participants in each group. The relationship between MTrPs and scapular impairment has been suggested because of the high prevalence of shoulder girdle muscles harboring MTrPs in people with unilateral, non-traumatic shoulder pain (Bron et al., 2011). Alterations of scapular kinematics, including decreased upward rotation and external rotation or posterior tilting of the scapula during arm elevation, might reduce the subacromial space. Decreased posterior tipping was more likely to occur in overhead athletes with UT MTrPs than in the controls in our study. In contrast, the upward rotation of the scapula was similar in the two groups and was close to the normal scapulohumeral rhythm during arm elevation (Johnson et al., 2001). Thus, our results support the theory that UT MTrPs are related to scapular posterior tipping impairment during arm elevation in overhead athletes.

Scapular posterior tipping impairment during arm elevation was most likely elicited by the scapular elevation fatigue task in the presence of UT MTrPs. These results are in agreement with the results from Tsai et al. (2003) who found decreased posterior tilting of the scapula during arm elevation after a repeated glenohumeral joint external rotation task (arm by side with elbow flexion of 90°). Additionally, decreased upward rotation and external rotation were also found during the task. Rich et al. (2016) also found a decrease in scapular upward rotation during arm elevation immediately after a functional fatigue task of serving tennis balls. However, increased upward rotation of the scapula was reported after fatigue tasks of repeated glenohumeral joint external rotation (Joshi et al., 2011) and repeated prone lifting (Chopp-Hurley and Dickerson, 2015). Noguchi et al. (2013) found that a repeated prone rowing protocol did not result in any significant changes in scapular movements. These alterations of scapular kinematics may depend on the features of the various tasks, including the plane of the movement, sustained or repetitive movement, voluntary or passive contraction, global or selective targeted muscles, and so on.

Regarding the influence of the UT MTrPs on muscular activity, our results indicated no consequences of inappropriate recruitment of the UT. The muscles hosting MTrPs may have increased responsiveness, delayed relaxation and increased fatigability in electromyography signals during voluntary muscle contraction (Donnelly et al., 2018). Instead of increased responsiveness in the UT, the finding of increased LT muscular activity in our results may indicate a compensatory mechanism for the UT dysfunction to maintain the force production of scapular movements during arm elevation. This may be related to satellite trigger point features described in a textbook about myofascial pain and dysfunction written by Simons et al. (1999). When the pain resulting from MTrPs becomes persistent, the patient may develop satellite trigger points, usually in an overloaded synergist muscle (Donnelly et al., 2018). The theory of MTrPs and associated synergist muscular activation requires further investigation.

The scapular elevation fatigue task in the current study revealed the same muscle fatigue patterns in both groups, for the UT and LT both developed muscle fatigue. These results rejected our second hypothesis, namely, that subjects with UT MTrPs would reveal a greater response to the fatigue task than would those without UT MTrPs. For the effects of the fatigue task on muscular activities, we found increased muscular activities of the UT and LT in arm lowering after the scapular elevation fatigue task. This increase in EMG amplitude is in accordance with the characteristics of fatigue (Enoka and Duchateau, 2008; Troiano et al., 2008; Umehara et al., 2018). In the literature, a shoulder external rotation fatigue task with dumbbell weight of 25% peak force was demonstrated to result in increased infraspinatus activity, decreased LT activity, and no change in the UT or SA (Joshi et al., 2011). The conflicting results on the LT might indicate that the function of the LT is task dependent, for it is a scapular upward rotator or stabilizer during a scapular elevation fatigue task or a shoulder external rotation fatigue task, respectively.

Limitations of this study should be noted. First, scapular kinematics beyond 120° were not analyzed in this study because of the inadequate reliability and validity of the measurement instruments. Therefore, changes in scapular kinematics beyond 120° remain unclear. Second, fatiguing exertion may have caused perspiration or changes in skin temperature and influenced the adhesiveness of the electrodes or skin markers. To minimize these unavoidable effects, the temperature of the room was maintained at 24°C and the fixation of sensors was bolstered with breathable paper tape during the experiment. Third, we only measured the outcomes without an additional load in the scapular plane. The effects of UT MTrPs and the isometric scapular elevation task on kinematic and muscular activity in the sagittal and coronal planes and under the loaded condition are still unknown. Lastly, participants recruited in the present study were limited to young amateurs in overhead sports with mild symptoms resulting from UT MTrPs. Therefore, the results may not be generalizable to other populations.

## Conclusions

The presence of upper trapezius myofascial trigger points in amateur overhead athletes is related to impaired scapular kinematics and associated muscular activities during arm elevation after a fatigue task. Decreased scapular tipping during 90 degrees of raising/lowering after a fatigue task may lead to impingement. The lower trapezius synergist muscle may be recruited as an upward rotator of the scapula to compensate for upper trapezius myofascial trigger points during arm elevation. For overhead athletes with UT MTrPs, strengthening of the lower trapezius and anterior shoulder stretching exercises are recommended.
